# Textile Materials for the Design of Wearable Antennas: A Survey

**DOI:** 10.3390/s121115841

**Published:** 2012-11-15

**Authors:** Rita Salvado, Caroline Loss, Ricardo Gonçalves, Pedro Pinho

**Affiliations:** 1 Unidade de Investigação Materiais Têxteis e Papeleiros, Universidade da Beira Interior, 6201-001 Covilhã, Portugal; E-Mail: caroline.loss@hotmail.com; 2 Instituto de Telecomunicações, Campus Universitário de Santiago, 3810-135 Aveiro, Portugal; E-Mails: rgoncalves@av.it.pt (R.G.); ptpinho@av.it.pt (P.P.)

**Keywords:** wearable antenna, flexible antenna, textile materials, dielectric properties

## Abstract

In the broad context of Wireless Body Sensor Networks for healthcare and pervasive applications, the design of wearable antennas offers the possibility of ubiquitous monitoring, communication and energy harvesting and storage. Specific requirements for wearable antennas are a planar structure and flexible construction materials. Several properties of the materials influence the behaviour of the antenna. For instance, the bandwidth and the efficiency of a planar microstrip antenna are mainly determined by the permittivity and the thickness of the substrate. The use of textiles in wearable antennas requires the characterization of their properties. Specific electrical conductive textiles are available on the market and have been successfully used. Ordinary textile fabrics have been used as substrates. However, little information can be found on the electromagnetic properties of regular textiles. Therefore this paper is mainly focused on the analysis of the dielectric properties of normal fabrics. In general, textiles present a very low dielectric constant that reduces the surface wave losses and increases the impedance bandwidth of the antenna. However, textile materials are constantly exchanging water molecules with the surroundings, which affects their electromagnetic properties. In addition, textile fabrics are porous, anisotropic and compressible materials whose thickness and density might change with low pressures. Therefore it is important to know how these characteristics influence the behaviour of the antenna in order to minimize unwanted effects. This paper presents a survey of the key points for the design and development of textile antennas, from the choice of the textile materials to the framing of the antenna. An analysis of the textile materials that have been used is also presented.

## 1. Introduction

Body worn systems endowed with sensing, processing, actuation, communication and energy harvesting and storage abilities are emerging as a solution to the challenges of ubiquitous monitoring of people in applications such as healthcare, lifestyle, protection and safety [1]. Accordingly, the new generation of clothing will be able to sense, communicate data and harvest energy in a nonintrusive way [1,2]. The wearable antenna is thus the bond that integrates cloth into the communication system, making electronic devices less obtrusive. To achieve good results, wearable antennas have to be thin, lightweight, low maintenance, robust, inexpensive and easily integrated in radio frequency (RF) circuits [3–6]. Thus, planar antennas are the preferred type of antenna as, despite the fact their maximum attainable bandwidth-efficiency is significantly lower than the theoretical limit for electrically small antennas, they allow an excellent integration of the antenna with the RF circuits, feeding lines and matching circuits on a standard multilayer board material [4]. Therefore, they might be integrated in cloth in a minimally intrusive way [2,5]. In particular, the microstrip patch antennas are good candidates for body-worn applications, as they mainly radiate perpendicularly to the planar structure and also their ground plane efficiently shields the body tissues [5,7]. Specific requirements for the design of wearable antennas are thus: planar structure; flexible conductive materials in the patch and ground plane; and flexible dielectric materials [3,5,8]. The characteristics of the materials are crucial for the behaviour of the antenna. For instance, the permittivity and the thickness of the dielectric substrate mainly determine the bandwidth and the efficiency performance of the planar antenna [4]. Also, the conductivity of the ground plane and of the patch is an important factor in the efficiency of the antenna and must be the highest possible.

Textile materials, being universally used and easily available, are possible materials to design wearable antennas for in- and on-Body Area Networks (BAN). The characterization of their electric and electromagnetic properties is essential for the design of the antenna [8]. Electrical properties of conductive textiles have been accurately characterized using the Transmission Line Method [9], Cavity Method [10] and MoM-segment Method [11], and their surface resistivity are often given by specialized producers. Therefore, specific conductive textiles, sometimes designated electrotextiles, that are commercially available have been successfully used in antennas [5,12–16]. Ordinary textile fabrics have been used as dielectric substrates. However, little information is found on the electromagnetic properties of regular commercialized textiles. Therefore, this paper is focused on the analysis of textile materials, mainly dielectric fabrics, that have been used in antennas [2,17–22].

The following section presents an overview of the influence of some features of the textile materials, dielectrics and conductive ones, in the behaviour of the antennas. It also gives some guidelines for the choice of materials for the design of textile antennas. Later, Section 3 reviews the textile materials that have been used to develop different wearable antennas, focusing on the regular fabrics used as dielectrics and listing their relative permittivity and loss tangent values. Furthermore, Section 4 presents an overview of guidelines for the construction of planar and wearable antennas and the techniques used to assemble the various materials. Finally, Section 5 presents the main conclusions of this work.

## 2. Important Features of Textile Materials in the Design of Wearable Antennas

Fabrics are planar fibrous materials which properties are mainly determined by the properties of the component fibres and the structure of the yarns and/or of the fabric. They are porous materials, in which the density of the fibres, air volume and size of the pores determine general behaviour, for instance, air permeability and thermal insulation. Accordingly, fabrics are flexible and compressible materials which thickness and density might change with low pressures. Moreover, the main orientation of the fibres and/or yarns introduces an intrinsic planar anisotropy of general properties. Plus, fibres are constantly exchanging water molecules with the surroundings, which affects their morphology and properties. All these features are somehow difficult to control in real applications of textiles, therefore it is important to know how they may influence the behaviour of the antenna in order to minimize any unwanted effects. Moreover, some characteristics of the textile materials known to influence the performance of the antenna and referred in the literature are reviewed in this section.

### 2.1. The Dielectric Constant (Relative Permittivity) of the Fabrics

The constitutive parameter of dielectrics is the permittivity, *ε*, that is a complex value parameter. It is usually expressed as a relative value *ε_r_*: *ε* = *ε_0_ε_r_* = *ε_0_*(*ε*′*_r_* − *jε*″*_r_*), where *ε_0_* is the permittivity of vacuum, which is 8.854 × 10^−12^ F/m [17]. In general, the dielectric properties depend on the frequency, temperature, and surface roughness [17], and also on the moisture content, purity and homogeneity of the material [18]. The real part of the relative permittivity, *ε*′*_r_*, is called the dielectric constant, but one must note that it is not constant in frequency. The ratio of the imaginary part to real part is called the loss tangent, tanδ = *ε*″*_r_*/ *ε*′*_r_*.

Some researchers have studied and reviewed the dielectric properties of textiles [19–21]. As textile materials are anisotropic materials, their characterization also depends on the electric field orientation. This anisotropy is fully described with a permittivity tensor, although in most practical applications like the ones surveyed in this paper, a specific component of this tensor is enough to characterize the behaviour of the textile material for a specific application. Thus, the relative permittivity describes the behaviour of the material tested under a specific electric field orientation and frequency.

The dielectric behaviour of textile materials depends on the properties of the constituent fibres and polymers [19], and on the fibre packing density in the fibrous material [18,20]. However, textile fabrics are rough, porous and heterogeneous, having air in between the fibres, making their characterization difficult [23]. In addition, the ability of the fibres to absorb moisture must also be considered in the characterization of the dielectric behaviour of textiles, as will be explained further in Section 2.4. Thus, the accurate measurement of dielectric characteristics of textiles is challenging and different experimental techniques have been used, such as the Cavity Perturbation Method [4,10], MoM-segment method [9], Resonance Method [21], Free Space Method [24] and Transmission Line Method [25–27]. Among these techniques, the simplest ones and thus very promising ones are the techniques based on the measurement of the behaviour of transmission lines [25–27].

In general, textiles present a very low dielectric constant as they are very porous materials and the presence of air approaches the relative permittivity to one. As an example, Table 1 shows the dielectric properties of normal textile fabrics, possible dielectric substrates, that were obtained with a waveguide cavity method, under 2.6 GHz [10].

The low dielectric constant reduces the surface wave losses which are tied to guided wave propagation within the substrates. Therefore, lowering the dielectric constant increases spatial waves and hence increases the impedance bandwidth of the antenna, allowing the development of antennas with acceptable efficiency and high gain [3,28–30]. Again, one should note that the relative permittivity value changes with the moisture content of the substrate affecting the bandwidth of the antenna [2,29].

### 2.2. Thickness of the Dielectric Fabrics

The bandwidth and efficiency performance of a planar microstrip antenna is mainly determined by the substrate dielectric constant and its thickness [4,31]. As referred before, the changes in permittivity may change the antenna bandwidth, but lowering the substrate permittivity can also increase the resonance frequency of the antenna.

As textile materials present a quite narrow range of permittivity values, it is therefore their thickness, which values may present much larger variations, that will mainly determine the bandwidth as well as the input impedance of the antenna and so its resonance frequency [28]. The thickness of the dielectric material is thus crucial in the design of antennas [2,31]. For a fixed relative permittivity, the substrate thickness may be chosen to maximize the bandwidth of the planar antenna. However, this value may not optimize the antenna efficiency. Therefore, the choice of the thickness of the dielectric material is a compromise between efficiency and bandwidth of the antenna [4,31].

The influence of the thickness on the bandwidth (BW) of the antenna may be explained by Equation (1), where *Q* is the antenna quality factor:

(1) BW~1/Q

The Q factor is influenced by the space wave (*Q_rad_*) losses, the conduction ohmic (*Q_c_*) losses, the surface waves (*Q_sw_*) and dielectric (*Q_d_*) losses as shown in Equation (2) [32]:

(2) $$\frac{1}{Q_{t}}=\frac{1}{Q_{\text{rad}}}+\frac{1}{Q_{c}}+\frac{1}{Q_{d}}+\frac{1}{Q_{\text{SW}}}$$

For thin substrates(*h* ≪ *λ*_0_) the quality factor associated with radiation (*Q_rad_*) is usually the dominant factor and is inversely proportional to the height of the substrate [32]. Therefore, increasing the height of the substrate lowers the Q factor (*Q_t_*). As the Q-factor decreases with an increased aperture between the patch and the ground planes of the antenna, a thicker substrate allows a larger antenna bandwidth [5].

Moreover, the thickness of the substrate also influences the geometric sizing of the antenna. This means that a thick substrate with low relative permittivity (value between 1 and 2) results in a large patch and a thin substrate with the same dielectric constant results in a smaller patch [5].

There are commercially available fabrics with a very diverse range of thickness values. Plus, nominal thickness values are given in any technical data sheet, allowing a careful choice of the material based on the required thickness. Moreover, accurate values of the thickness of fabrics under specified pressure are easily obtained by simple standard methods, such as ISO 5084:1996 and ASTM D374-99(2004) or with a Digimatic Indicator [31]. Therefore, the thickness of the fabrics is a feature that may guide the search for suitable textile dielectrics.

### 2.3. The Electrical Surface Resistivity of the Conductive Fabrics

Fabrics are planar materials and therefore their electrical behaviour may be quantified by the surface resistance and characterized by the surface resistivity. The surface resistance, which unit is (Ω), is the ratio of a DC voltage to the current flowing between electrodes of specific configuration that are in contact with the same face of the material under test [33]. The surface resistivity is the ratio of the DC voltage drop per unit length to the surface current per unit width. Surface resistivity is thus a property of the material, not depending on the configuration of the electrodes used for the measurement. It is usually expressed in Ohm/square (Ω/sq; Ω/□ [34].

Despite the existence of several standard methods, *i.e.*,

- AATCC Test Method 76-2011: Electrical Surface Resistivity of Fabrics
- ASTM D4496: 2004 Standard Test Method for D C Resistance or Conductance of Moderately Conductive Materials
- ISO 10965:2011: Textile floor coverings—Determination of electrical resistance
- ISO 21178:2005: Light conveyor belts—Determination of electrical resistance

to measure the surface resistance and resistivity of textile fabrics, they are dedicated to moderately conductive materials and are still aim of analysis [35]. An accurate characterization of highly conductive fabrics demands other techniques, such as for instance the ones based on transmission lines and waveguide cavities [9–11,36].

For the antenna design, the relevant parameter is the conductivity of the fabric, *σ*, which unit is Siemens per meter (S/m). It is related to the surface resistivity, *ρ_s_*, by Equation (3), where *t* is the thickness of the fabric:

(3) $$\sigma =1/\left(\rho_{s}\cdot t\right)$$

Besides the dielectric constant of the substrate, the choice of the conductive fabric for the patch and the ground planes is also very important to assure a good performance of the antenna. In general these fabrics must have a very low electrical surface resistance in order to minimize the electric losses and thus increase the antenna efficiency. Despite the fact that the surface resistance value should be constant over the area of the antenna [2], the fabric may present some heterogeneities, such as for instance some discontinuities in the electric current. If these discontinuities are parallel to the surface current they will not interfere with the electromagnetic fields [37], but if discontinuities impede the flow of the electrical current, the fabric resistance will increase [2]. The structure of the fabric should thus be considered, seeking to determine the density, the continuity and the alignment of the conductive components, which may be fibres, filaments or surface covers. In [2] the studied fabrics having conductive fibres performed better than the coated fabrics, because of discontinuities that increased the surface resistance of the coated fabrics. In [10] higher conductive thread density in the woven fabric results in higher effective conductivity.

Wovens and knits with electric surface resistivity below 1 Ω/□ are commercially available (e.g., Less EMF Inc. at http://www.lessemf.com or Shieldex Trading at http://www.shielextrading.net) and have been successfully used [2,24,26]. However, knits may present higher anisotropy than wovens, showing different electric surface resistance along the longitudinal and the transversal directions and this anisotropy may increase with the knit deformation. Indeed, in fabrics composed of conductive threads, conductive paths exist in all directions through the conductive threads and/or inter-contact points across threads [10]. According to Locher *et al.* [2], for the studied knit material that is composed of silver plated polyamide fibres, deformations under 8% of elongation along the direction of the wales slightly change the surface resistivity. However, when elongating the knitted fabric along the direction of the courses the electric surface resistivity results stable up to 3% of elongation but then increases and at 8% of elongation it reaches triple the initial value.

**Figure 1.**
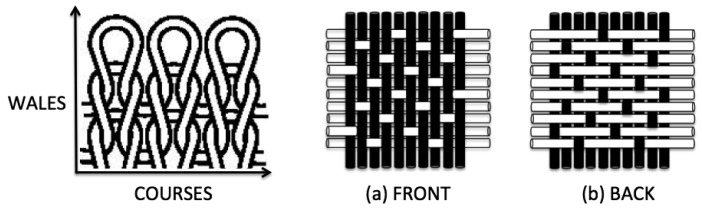
Schemes of: (left) Jersey knit; (middle and right) satin 5 woven; (a) front and (b) back.

The influence of the structure of the fabrics on the surface resistivity may be better understood looking at Figure 1 that shows schemes of regular patterns of fabrics: the jersey knit and the satin 5 weave patterns. If the conductive threads in the weave pattern are along the intended direction for current flow, woven patterns are much more efficient in terms of electrical conduction than knit patterns, because the conductive paths in woven are better aligned with the current direction, which minimizes the conductive losses, as concluded in [10].

Moreover, fabrics present differences between their faces that should be consider when positioning the conductive fabric in a planar assemblage. For instance, in Figure 1 the right and back sides of the satin 5 woven are clearly different. In [10], a satin 5 woven composed of conductive yarns (represented by white rods in Figure 1) and non-conductive yarns (represented by black rods) was tested in a microstrip resonator measurement to study the effect of the differences among faces on the electrical properties of planar structures, as wearable antennas. It was proved that keeping the conductive face against the dielectric substrate is preferred for lowering the electrical losses.

### 2.4. The Moisture Content of the Fabrics

Textiles always establish a dynamic equilibrium with the temperature and humidity of the air surroundings they are in contact with, as the fibres are constantly exchanging water molecules with the air. However, the amount of water that a material takes until reaching this equilibrium depends on the type of material. The extent to which a material is sensitive to moisture is described by its regain, which is defined, by the ratio of the mass of absorbed water in specimen to the mass of dry specimen, expressed as a percentage, [20]. In [20], page 169 shows the relation between regain and relative humidity of the air (RH), for various textile fibres, compiling studies made by several authors. Indeed, for the same RH conditions, there are textile fibres with largely different moisture contents. For instance, at 65% RH, wool fibre might present a regain of 14.5%, cotton might present a regain of 7.5% and polyester fibre might present a regain of 0.2% [20].

In general, the moisture absorption changes the properties of the fabrics. Because of this, textile metrology is performed always in a conditioned environment (20 °C and 65% RH). Also, for commercial transactions there are national legislations setting nominal values of moisture content that are values close to the regain obtained at 65% relative humidity.

Water has a dielectric constant of *ε*′*_r_* = 78 at 2.45 GHz and 25 °C [31]. Although this value depends on the salinity, temperature and frequency [38], water has a much higher and more stable dielectric constant than textile fabrics, whose dielectric constant is generally in the range *ε*′*_r_* = 1 – 2 because of their high porosity, as previously presented. Therefore, when water is absorbed by the textile fibres or is trapped into the fabric structure, it changes the electromagnetic properties of the fabric, increasing its dielectric constant and loss [2,5,19,20,31]. Charts presenting the relationship between the RH of the air or the moisture content of various fibres and their dielectric properties can be found in [20]. Additionally, several authors have been correlating the electromagnetic properties of fabrics to their absorption properties [31,38].

Likewise, the absorbed water by or trapped into the textile components of the antenna dramatically changes the behaviour of the antenna. The higher permittivity of the water drives the performance of the antenna, reducing its resonance frequency [2,6] and bandwidth [9,29,31].

Beyond these effects, when textile fibres absorb water they swell transversely and axially, causing tightening of the fabrics [20,39]. This tightness affects the dimensional stability of the fabrics and therefore affects the dimensional stability of the antenna, influencing its behaviour [2]. The swelling of the fibres also contributes to the change of the dielectric properties, as swollen fibres decrease the porosity of the fabric [39]. Indeed, as seen above, the low relative permittivity values of the fabrics are mainly determined by the presence of air in them. In [20] (page 227), experimental results from several authors are compiled, showing that values of swelling in water vary the same way as the values of the regain of the fibres. For instance, when cotton is in water, its cross section area might swell 40%, whilst polyamide only swells 3.2% [20].

Moreover, water absorption by the fibres is an exothermic reaction and the released heat of wetting is the greatest for the highly absorbing fibres. This affects the temperature and thus the electromagnetic properties of the material. More details and data quantifying these effects can be found in [20].

Therefore, climatic changes altering the relative humidity of the air or environmental water, ice and snow conditions will influence the textile antenna performance [31,40]. In the same way, when the antenna is used close to the skin, the hydrophobicity and the regain of the fabric are very important features [28,41] as the fabric will absorb moisture from the skin.

In general, the characteristics of antennas based on textile materials with small moisture absorption values (regain less than 3%) are more stable [31]. Therefore, materials with low regain are preferable for use as substrates and the same conclusion applies to the textile conductive components of the antenna.

### 2.5. Mechanical Deformations of the Dielectric and Conductive Fabrics

A curvature on a human body consists of a superposition of bends in arbitrary directions. Because of their excellent flexibility and elasticity textile materials adapt well to these surfaces. However, when the textile fabric adapts to the surface topology it bends and deforms, causing changes to its electromagnetic properties and thus influencing the antenna performance [2,29,42]. Indeed, the bending and the elongation of the dielectric fabric influences its permittivity and its thickness, which affects the resonance frequency of the antenna and especially the bandwidth, as previously explained [28].

Moreover, the variation of the geometric dimensions of the textile components of the antenna due to their elongation and/or compression diminishes the geometric precision of the shape of the antenna affecting its behaviour, which may change its resonance frequency [28].

In addition, from the point of view of manufacturing antennas, the elasticity of the fabrics is an inconvenience as it makes difficult the precise definition and cut of the shape of the components and also makes difficult the superposition of the several materials without folds. Wovens and nonwovens, being more stable fabrics than knits, allow higher geometrical accuracies of the frame of the antenna. In general, the accuracy depends on the thickness of the component yarns or fibres. For instance, conductive woven may allow an accuracy of about ±0.15 mm [2]. For these reasons knits are not stable enough for the dielectric of an antenna. However, as further presented in Section 4, they might be suitably stabilized if assembled with a more rigid textile material, such as a fabric with high tensile strength.

## 3. Textile Materials Used in Wearable Antennas

Wearable antennas are a recent research subject, although one of the first proposals on the subject appeared in 2001 [3], when Salonen *et al.* [22], presented a Planar Inverted F Antenna (PIFA) for dual-band operation, built on a flexible unspecified substrate. It was intended to be placed in the sleeve of clothing and operate at GSM (900 MHz) and Bluetooth (2.4 GHz) frequency, although the lower band was not achieved, the antenna still showed good performance, even for human-body presence, around the 2.4 GHz band. Later in 2003, they presented an antenna built on a textile substrate intended for WLAN applications [43], where results are claimed to be acceptable.

Furthermore, in 2004 Salonen *et al.* [28] proposed a GPS antenna with circular polarization, in which they have experimented with five different synthetic fabric materials as dielectric substrates. The conductive parts were made of copper tape. The dielectric synthetic materials sued were: (1) Vellux^®^, which is a 5 mm thick fabric covered on both surfaces with thin layers of plastic foam; (2) synthetic felt, which is a 4 mm thick nonwoven in which fibres are looser on the surface than in the centre; (3) Delinova 200^®^, which is a strong fabric made of polyamide Cordura^®^ fibres laminated with Gore-Tex membrane, weighing about 370 g/m^2^ and having a thickness of 0.5 mm; (4) fleece, which is a very soft polyester fabric with 4 mm thickness, commonly used in sportswear; (5) upholstery fabric, which is composed of three fabric layers bound together resulting in a thin (1.1 mm) fabric of polyester and acrylic that has firmness. The relative permittivity, ε′_r_, of the five fabrics was measured by a cavity perturbation method, at 1.575 GHz, and the values ranged between 1.1 and 1.7. Among the studied fabrics, the one made of high tenacity polyamide fibres (Cordura^®^) was pointed out as the more interesting fabric for the development of a flexible antenna, because of its constant thickness and its high resistance. These properties allow more stable geometric dimensions of the antenna.

More recent demonstrations on wearable antennas for Personal Area Networks (PANs) to operate in the 2.45 GHz Industrial, Scientific and Medical (ISM) band and for GPS applications are presented in [5,12]. In these examples, antennas for wearable protective clothing intended for professional use under rough conditions are presented and their behaviour in various practical scenarios is discussed. High performance aramid fabric that can withstand high temperatures is used as substrate while conductive textiles, like *Shieldit*^®^ and *FlecTron*^®^, are used for the antenna patch and ground plane. These antennas have shown acceptable performance, even in a real environment with human-body presence and when subjected to bending and deformations.

Locher *et al.* [2] have built four purely textile wearable patch antennas for Bluetooth applications. They have used three electrical conductive fabrics: (1) a nickel-plated woven fabric (with plating thickness about 250 nm applied on the fabric surface); (2) a silver-plated knitted fabric; (3) a silver-copper-nickel-plated woven fabric. Fabric (3) is the one preferred for building textile antennas with geometric precision, as it is woven and not knitted and its electric surface resistance was more homogeneous than the one of fabric (1). For the dielectric substrate, they used two types of fabrics: (1) woollen felt of 1,050 g/m^2^ with a thickness of 3.5 mm and (2) polyamide spacer fabric, of 530 g/m^2^, with a thickness of 6 mm. The felt was dimensionally more stable and harder to bend, whereas the spacer fabric was lighter and more elastic due to its knitting-based structure. The dielectric properties were measured by a transmission line method, at a frequency of 2.4 GHz, obtaining as results for the felt: permittivity *ε*′*_r_* = 1.45 and loss tangent *tan*δ = 0.02, and for the spacer fabric: permittivity *ε*′*_r_* = 1.14 and the loss tangent was negligible. The four different antennas produced have shown good performance and could satisfy the Bluetooth specifications, even when subjected to bending effects. However the antennas lose their circular polarization when subject to bending.

The same year, Tronquo *et al.* [13] presented rectangular-ring textile antennas for body area networks (BAN) that are circularly polarized, covering a bandwidth of more than 190 MHz. For the conductive antenna patch and ground plane they used the conductive fabric named *Flectron*^®^, that is a thin copper plated fabric with low surface resistivity, lesser then 0.1 Ω/□ For the dielectric substrate they used a fleece fabric of 2.56 mm thickness. Its dielectric properties were measured testing antennas and they obtained a relative permittivity of *ε*′*_r_* = 1.25.

In 2007, Zhu and Langley [18] developed a dual-band coplanar patch antenna integrating electromagnetic bandgap material (EGB), to operate at the 2.45 and 5.8 GHz wireless bands. The conductive parts were made of *Zelt*^®^ fabric while the dielectric substrate was a thin felt, with 1,1 mm thickness, and with relative permittivity *ε*′*_r_* = 1.30 and tanδ = 0.02.

Matthews and Pettitt presented in [44] three types of antennas which are integrated into clothing, a broadband wire dipole, a bowtie and a spiral antenna, operating in frequencies from 100 MHz to 1 GHz. They have tested different materials (textiles and others), different frames and manufacturing techniques. Among the tested conductive materials there are conductive ribbon, conductive paint and ink, conductive nylon fabric (that is also adhesive on the back face), phosphor bronze mesh fabric (also adhesive on the back face), conductive thread, liquid crystal polymer (LCP) and copper coated fabric. The phosphor bronze mesh, LCP and copper coated fabric have the advantage that the antennas can be directly soldered to. In some antennas, a conducting epoxy was used to bond materials, but this shows some lack of robustness. In terms of radio frequency (RF) performance of the designed antennas, the spiral antenna, in which the spiral is broidered with conductive thread, performed worse than any other antenna and was clearly loss. Overall, based on RF performance, the most attractive materials to design wearable antennas were the textile fabrics: the conductive nylon and the copper coated fabrics.

In [16] the stability and efficiency of wearable and washable antennas are discussed for textile antennas in which the conductive parts were screen-printed with conductive ink. These antennas have shown acceptable performance. The combination of screen-printing with a breathable thermoplastic polyurethane (TPU) coating assured the performance was maintained even after several wash cycles. An embroidered technique was used in [45,46] to sew conductive fibres into polymer and fabric substrates, and it was proved that by increasing the density of the embroidering stitching, the conductivity of the conductive section is increased and also the accuracy of fabricated prototypes, which allowed better conformance with the simulations. Dipole, spiral and microstrip patch antennas were fabricated with this technique and presented very good RF performance when compared to the corresponding rigid copper structures. Moreover, these antennas when made on flexible polymer substrates conform well to curved surfaces and thus maintain their performance. Table 2 summarizes the main characteristics of the textile materials that have been used to develop wearable antennas, focusing mainly in the dielectric materials.

The performance of the textile antennas presented earlier can be improved with integrated solutions, for instance, if diversity techniques such as Multiple-Input Multiple Output (MIMO) are considered [47], or, as shown in [48], with the introduction of a low-noise amplifier (LNA) in a wearable garment which was used to achieve an active integrated antenna, increasing the sensitivity and the gain of the overall system.

Declercq *et al.* [13] showed another integrated solution consisting of an aperture-coupled antenna on a textile and foam substrate, with a flexible solar cell, for tracking and monitoring solutions. Instead of integrating a LNA to increase the wearable antenna performance, Zhu and Langley [11,12] developed a dual-band coplanar patch antenna, to operate in the 2.45 and 5.8 GHz wireless bands, in which they integrated an electromagnetic band gap (EBG) to reduce body-presence effects and increase antenna gain. As shown in Table 2, the conductive parts were made of *Zelt*^®^ fabric while the dielectric substrate was a thin felt with relative permittivity *ε*′*_r_* = 1.30 and tanδ = 0.02. They proved that the introduction of the 3 × 3 array EBG with the coplanar patch could reduce the radiation towards the body by 10 dB, while increasing the antenna gain in 3 dB.

## 4. Construction of Wearable Antennas

After choosing the textile materials to design an antenna, their assemblage in the antenna is also crucial and specific, as they are very deformable materials. Thus, the conformation of the conductive patch with the dielectric substrate is critical [2]. Many authors have been improving the manufacturing processes [2,5,24,27] to construct textile antennas and some guidelines can be summarized as follows:

(1) The geometrical dimensions of the patch should remain stable while connecting to the dielectric substrate as the mechanical stabilization of both materials is essential to preserve the desired antenna characteristics [2,31]. In the microstrip patch antennas developed by Hertleer *et al.* [31] an alteration of no more than 0.5 mm on the length or the width of the patch influenced the performance of the antenna by causing a slight shift of the antenna characteristics. For this reason, woven fabrics, being more stable, are preferred to make the patches. However, the antenna geometrical stability can be achieved if at least one component is less deformable. For example, bonding using an adhesive sheet with a deformable patch, such as a conductive knit, with a less deformable substrate, such a woven dielectric, results in a stable frame [2,28].
(2) The techniques used to connect the various layers must not affect the electrical properties of the patch, such as its surface resistivity, nor the properties of the substrate. Connections using adhesive sheets or conductive fabrics with a thermal adhesive face have shown good results [2,5,29,31]. Indeed, the adhesive remains at the interface of the materials and therefore the surface resistance of the patch and the relative permittivity of the substrate are not significantly changed. However, in [29] the authors show that the adhesive layer introduces extra losses in the substrate. This process of attachment of the superposed layers is very simple to perform by a simple ironing operation. However, attention should be made to the ironing process, in special if the patch is made of a fabric with metallic components. Indeed, the oxidation of the metallic component, due to the hot moistening of the fabric, may increase the surface resistance of the fabric and so decrease the efficiency of the textile antenna [27].
  Connection with seams is an alternative technique [7,31] but it presents some difficulties. Firstly, the seam must be plane, without wrinkling, what might be difficult to achieve with deformable materials. Secondly, the stitch passes through all materials: the patch, then the substrate and further the ground plane of the antenna, which may cause electrical shorts between them. In Locher *et al.* [2] report that the sewing needle has pulled conductive fibres from the patch through the substrate, shorting the patch with the ground plane.
  Another technique is connecting with liquid adhesives [31]. However, it is difficult to apply a thin layer of glue. This difficulty introduces heterogeneity and in the zones where there are accumulations the glue may play the role of insulator between the conductive yarns of the patch. Furthermore, these adhesives are usually stiff and brittle, and so they cannot be applied in an area-wide manner on textiles as they will interfere in their flexibility [2]. In order to obtain a uniform thickness of the attachment of the several layers, Tronquo *et al.* [29,31] perform an additional stitch, in addition to the glue.
  In [5] a smooth fabric was added to both faces of the substrate to optimise the attachment of the conductive components.

(3) The positioning of the textile components must consider the differences between right and back faces, in terms of roughness and of density of conductive elements [7,10]. In [10], a satin 5 woven was tested in a microstrip resonator, placing it in two positions: (1) with the right face against the dielectric substrate and (2) with the back face, the conductive one, against the dielectric substrate. It was observed that when the conductive face is placed on the top of the substrate and so underneath the nonconductive yarns of the nonconductive face, most of the electrical field is contained in the substrate. Thus, the dielectric loss in the nonconductive yarns is minimized.
(4) The core of the antenna may be obtained by stacking low-loss fabrics [7], adjusting this way the desired thickness of the substrate. However this introduces heterogeneities in the substrate due to the extra layers of air between the fabrics, influencing its dielectric properties.
(5) Finally, the connections at the antenna terminals may also be critical as in wearable and flexible antennas these connections have to be mechanically robust. In general, textile fabrics cannot be directly soldered to (an exception is *Flectron*^®^ that already showed good resistance to soldering [5]). Therefore conductive epoxy has been used, but some concerns remain as this connection is not very resistant [44].

## 5. Conclusions

The developed wearable antennas are mainly planar ones, specifically microstrip patch antennas, because they mainly radiate perpendicularly to the planar structure and also their ground plane efficiently shields the human body. The bandwidth and efficiency performance of a planar microstrip antenna is mainly determined by the substrate dielectric constant and its thickness.

In general, textiles present a very low dielectric constant, between 1 and 2, as they are very porous materials and the presence of air approaches the relative permittivity to one. The low dielectric constant reduces the surface wave losses that are tied to guided wave propagation within the substrates. Therefore, lowering the dielectric constant increases spatial waves and hence increases the impedance bandwidth of the antenna. However, lowering the substrate permittivity can also increase the resonance frequency of the antenna, allowing the development of antennas with acceptable efficiency and high gain.

In addition, the ability of the fibres to absorb moisture must also be considered in the characterization of the dielectric behaviour of textiles. Water has a much higher and more stable dielectric constant than textile fabrics. Therefore, when water is absorbed by the textile fibres or is trapped in the fabric structure, it changes the electromagnetic properties of the fabric, increasing its dielectric constant and loss tangent. Likewise, the absorbed water by or trapped into the textile substrate reduces the resonance frequency and bandwidth of the antenna. In general, the characteristics of antennas based on textile materials with small moisture absorption (regain less than 3%) are more stable. Therefore, materials with such low regain values are preferable for use as substrates and as conductive components of the antenna.

The thickness of the dielectric material is also crucial in the design of antennas. For a fixed relative permittivity, the substrate thickness may be chosen to maximize the bandwidth of the planar antenna. However, this value may not optimize the antenna efficiency. Therefore, the choice of the thickness of the dielectric material is a compromise between efficiency and bandwidth of the antenna. Moreover, the thickness of the substrate also influences the geometric sizing of the antenna.

The conductive fabrics for the patch and the ground planes must have a very low electrical surface resistivity in order to minimize the electric losses and so increase the antenna efficiency. There are several conductive textile fabrics, and also yarns, available on the market that has been successfully used in planar antennas. Coated fabrics might perform worse than the fabrics having conductive fibres, because of discontinuities that may increase the surface resistivity. Wovens and nonwovens, being more stable fabrics than knits, allow higher geometrical accuracies of the frame of the antenna and thus may be preferred to make the patch. In general, the sizing accuracy of the patch made of woven fabric depends on the thickness of the component yarns.

After choosing the textile materials, their assemblage may also be critical, as the elongation and bending causes mechanical deformations that interfere with the antenna behaviour. The presence of at least one textile material presenting high tensile strength, high bending rigidity and stable geometry stabilizes the frame of the antenna. When connecting the various layers making up the antenna, the positioning of the textile fabrics must consider differences between right and back faces in terms of roughness and of density of conductive elements, in order to minimize losses.

Finally, despite the fact the performance of the textile antennas can be improved with integrated solutions and despite the major progresses already achieved in the development of wearable antennas, further study and better characterization of ordinary and conductive textile materials may still offer relevant improvements to their design and to the optimization of their behaviour.

## Figures and Tables

**Table 1.** Dielectric Properties of normal fabrics tested in [10].

**Nonconductive Fabric**	***ε***′*_r_*	**tanδ**
Cordura^®^	1.90	0.0098
Cotton	1.60	0.0400
100% Polyester	1.90	0.0045
Quartzel^®^ Fabric	1.95	0.0004
Cordura/Lycra^®^	1.50	0.0093

**Table 2.** Comparison of the textile materials used to design wearable antennas.

**Reference**	**Application**	**Dielectric material**				**Conductive material**	**Performance**
		**Material**	***h*(mm)**	***ε****_r_*	**tan δ**		
[22]	GSM (900 MHz) and Bluetooth (2.4 GHz)	Unspecified material	0.236	3.29	0.0004	-	Acceptable
[43]	WLAN (2.4 GHz)	Fleece fabric	3	1.04	-	Knitted copper fabric	Acceptable
[28]	GPS (1.5 GHz)	*Cordura*^®^	0.5	Between 1.1 and 1.7	-	Copper tape	Good
[5,12]	ISM (2.4 GHz) and GPS (1.5 GHz)	Fleece fabric	2.56	1.25	-	*Flectron*	Acceptable to Good
[13]	ISM (900 MHz)	Polyurethane protective Foam	11	1.16	0.01	*Flectron*	Acceptable
[14,15]	WLAN (2.4 GHz and 5.8 GHz)	Felt	1.1	1.30	0.02	*Zelt*	Acceptable
[16]	ISM (2.4 GHz)	Cotton/Polyester	2.808	1.6	0.02	*Flectron*/Conductive ink	Acceptable
[6,46]	Not specific (2–2.4 GHz)	Polydimethylsiloxane (PDMS)	-	3.0–13	0.02	Embroidered conductive fibres	Good
[2]	Bluetooth (2.4 GHz)	Polyamide spacer fabric	6	1.14	Negligible	Silver-copper-nickel plated woven fabric	Good
[2]	Bluetooth (2.4 GHz)	Woollen felt	3.5	1.45	0.02	Silver-copper-nickel plated woven fabric	Good
